# The Value of Longitudinal Research and the Contribution of the Canadian Armed Forces Members and Veterans Mental Health Follow-up Survey

**DOI:** 10.1177/07067437211019666

**Published:** 2021-05-26

**Authors:** Nicola T. Fear

**Affiliations:** 1 4616 King’s College London, Weston Education Centre, London, United Kingdom

**Keywords:** cohort, military, veteran, mental health, methodology

All researchers know the importance of ensuring any study you conduct is methodologically robust, with different studies being more appropriate to answer certain research questions and different types of study being more challenging to conduct than others. Cross-sectional studies although useful can only tell us about the state of play at that time, while longitudinal studies allow us to explore changes over time and separate cause and effect. Longitudinal (or cohort) studies are established to follow up over time a group of people who share a particular “exposure” or characteristic. There are several extremely well-known types of cohort studies, for example, birth cohorts (i.e., the Dunedin Multi-disciplinary Health and Development Study^1^) and occupational cohorts (i.e., the Whitehall study^2^). Although they can provide rich data, they can be expensive, time-consuming and logistically challenging—especially minimising attrition at each follow-up point.

Questions are often asked about the mental health, physical health, and wellbeing of military personnel—especially regarding the longer-term impact of deployment and the transition from military to civilian life. In order to address these questions, it is essential that longitudinal studies are undertaken—these studies can explore the changing experiences of service personnel and veterans.^3^ To my delight, in this special issue of the *Canadian Journal of Psychiatry*, there are 6 papers from Sareen and his team reporting on the Canadian Armed Forces (CAF) Members and Veterans Mental Health Follow-Up Survey.^4–9^ Sareen et al. followed up their baseline cohort (established in 2002) 16 years later (in 2018), and in this special issue, we get to see how things have changed for Canadian military personnel over this time.

Two of these 6 papers are focused on methodology^8,9^—detailing what they did, the challenges they faced, and the resultant follow-up sample. Achieving an adequate follow rate is essential for cohort studies, overall 2,941 members of the original cohort (*n* = 5,155) were followed up and re-interviewed. This is a major achievement given the 16 years that have elapsed since the baseline. At follow up, 66% of participants were veterans, 88% were male with a mean age of 51 years. The key factors associated with attrition included age (those of younger age being less likely to respond), rank (those holding lower ranks being less likely to respond), and previous suicide attempt (individuals with a previous suicide attempt were less likely to respond). All subsequent analyses were weighted to take this potential response bias into account. Reassuringly, the key factors of interest (including mental health status at baseline) were not associated with attrition. Other military cohort studies use self-complete online or postal surveys (e.g., Stevelink et al.^3^); here, data were collected in person using a computer-assisted personal interview with most interviews taking place in the respondents’ homes, conducted by lay interviewers. This may have limited participation but does enable the use of more sophisticated data collection tools and measures. However, asking participants to recall experiences that occurred over the past 16 years is likely to be difficult for some and will introduce bias.

What do we learn from this series of papers? I suggest you read the papers as they are filled will lots of results, and I am only going to give you some highlights! The key findings that interested me were around the increases seen in past year prevalence of any mood or anxiety disorder (increased from 12% [in 2002] to 28% [in 2018]), and for PTSD (increased from 3% [in 2002] to 10% [in 2018]) and comorbidity (having three or more conditions; increased from 2% [in 2002] to 11% [in 2018]).^7^ These results indicate an increasing burden of mental health disorders among the CAF and also on the health care system to effectively identify and treat these individuals. There was a high level of reporting of childhood maltreatment, which was associated with adverse mental health.^3^ Along with a cumulative impact of childhood maltreatment and deployment related traumatic events on mental health.^3^ Equivalent findings have been reported by others, but I was surprised that almost two thirds of participants reported experiencing child maltreatment—some of these data were collected in 2002 and expanded on in 2018. Could recall bias play a role here?

Overall, this series of papers provides robust evidence which has implications for health care providers, policy makers, Canadian military/veteran health researchers, and the CAF community. Further, although there are situational and cultural differences between Canada and other nations, these results are important for the international community and add to the growing body of evidence regarding the mental health consequences of military service and deployment.

The 6 papers published here have only just started to scratch the surface of the data available from the CAF Members and Veterans Mental Health Follow-Up Survey, with many other topics waiting to be explored. It is wonderful to see that the data are available via Statistics Canada Research Data Centres, which will enable researchers across Canada to explore and answer novel questions. Providing greater access to these data will ensure that this study will continue to provide evidence for many years.

When embarking on a research study—I’d like to encourage researchers to be brave and aim to establish a cohort study. As we can see from the work of Sareen et al. revisiting the same cohort 16 years later provides a wealth of information and evidence. What next for the CAF Members and Veterans Mental Health Follow-Up Survey? Can we encourage Sareen et al. to consider another follow up? In another 16 years, the average age of the participants will be 67 years of age and entering “older” age with potentially increasing health needs. I shall await with keen interest!

## References

[1] Poulton R Moffitt TE Silva PA . The Dunedin multidisciplinary health and development study: overview of the first 40 years, with an eye to the future. Soc Psychiatry Psychiatr Epidemiol. 2015;50(5):679–693. doi: 10.1007/s00127-015-1048-8. 25835958 10.1007/s00127-015-1048-8 PMC4412685

[2] Marmot M Brunner E . Cohort profile: the Whitehall II study. Int J Epidemiol. 2005;34(2):251–256. doi: 10.1093/ije/dyh372. 15576467 10.1093/ije/dyh372

[3] Stevelink S Jones M Hull L , et al. Mental health outcomes at the end of the British involvement in the Iraq and Afghanistan conflicts: a cohort study. Br J Psych. 2018;213(6):690–697. doi: 10.1192/bjp.2018.175. 10.1192/bjp.2018.175 PMC6429255 30295216

[4] Afifi T Sareen J Taillieu T , et al. Association of child maltreatment and deployment-related traumatic experiences with mental disorders in active duty service members and veterans of the Canadian armed forces. Can J Psychiatry. 2021. doi: 10.1177/0706743720987086. 10.1177/0706743720987086 PMC8649822 33472392

[bibr5-07067437211019666] Enns MW Mota N Afifi TO , et al. Course and predictors of major depressive disorder in the Canadian armed forces members and veterans mental health follow-up survey. Can J Psychiatry. 2021. doi: 10.1177/0706743720984677. 10.1177/0706743720984677 PMC8649828 33406886

[bibr6-07067437211019666] Mota N Bolton SL Enns MW , et al. Course and predictors of posttraumatic stress disorder in the Canadian armed forces: a nationally representative, 16-year follow-up study. Can J Psychiatry. 2021. doi: 10.1177/0706743721989167. 10.1177/0706743721989167 PMC8649830 33522288

[bibr7-07067437211019666] Sareen J Bolton SL Mota N , et al. Lifetime prevalence and comorbidity of mental disorders in the two-wave 2002-2018 Canadian armed forces members and veterans mental health follow-up survey (CAFVMHS). Can J Psychiatry. 2021. doi: 10.1177/07067437211000636. 10.1177/07067437211002697 PMC8649824 33739174

[bibr8-07067437211019666] Bolton SL Afifi TO Mota N , et al. The importance of examining attrition in the Canadian armed forces members and veterans mental health follow-up survey (CAFVMHS). Can J Psychiatry. 2021. doi: 10.1177/07067437211002697. 10.1177/07067437211002697 PMC8649824 33739174

[9] Afifi TO Bolton SL Mota N , et al. Rationale and methodology of the 2018 Canadian armed forces members and veterans mental health follow-up survey (CAFVMHS): a 16-year follow-up survey. Can J Psychiatry. 2021.doi: 10.1177/0706743720974837. 10.1177/07067437211002697 PMC8649824 33739174

